# Suicidality Risks Associated with Finasteride, a 5-Alpha Reductase Inhibitor: An Evaluation of Real-World Data from the FDA Adverse Event Reports

**DOI:** 10.3390/ph18070957

**Published:** 2025-06-25

**Authors:** Hilal A. Thaibah, Otilia J. F. Banji, David Banji, Hadi A. Almansour, Thamir M. Alshammari

**Affiliations:** 1 Department of Clinical Practice, College of Pharmacy, Jazan University, Jazan 45142, Saudi Arabia; hthaibah@jazanu.edu.sa (H.A.T.); obanji@jazanu.edu.sa (O.J.F.B.); halmansour@jazanu.edu.sa (H.A.A.); 2 Pharmacy Practice Research Unit, College of Pharmacy, Jazan University, Jazan 45142, Saudi Arabia; 3 Department of Pharmacology & Toxicology, College of Pharmacy, Jazan University, Jazan 45142, Saudi Arabia; davidbani@jazanu.edu.sa

**Keywords:** finasteride, neurosteroids, pharmacovigilance, psychiatric adverse effects, suicidality

## Abstract

**Background**: Finasteride, a 5α-reductase inhibitor, is used for androgenetic alopecia and benign prostatic hyperplasia. However, concerns have emerged about its psychiatric side effects, including suicidality. This study analyzed finasteride-related reports from the FDA Adverse Event Reporting System (FAERS) to identify potential safety signals. **Methods**: Adverse events reported from 2015 to 2024 were extracted using preferred terms, quantified using Bayesian analysis and disproportionality metrics, including empirical Bayesian geometric mean (EBGM), information component (IC), reporting odds ratio (ROR), and proportional reporting ratio (PRR). **Results**: Most were male (87%), with 43% aged 18–40 years, primarily using finasteride for hair loss. Disproportionality metrics for suicidality-related events fluctuated between 2019 and 2024. In 2019, the ROR was 27.51 (95% CI: 23.22–32.58), the PRR was 21.96 (95% CI: 18.54–26.01), the EBGM was 20.50, and the IC was 4.36. A slight decline was observed in 2020, a surge in 2021, and a peak in 2022 (ROR 34.64 (95% CI: 28.36–41.88), PRR 27.82 (95% CI: 22.30–34.61), EBGM 24.96, IC 4.64). Although a sharp rise in suicidality reports was noted in 2024, the rates of ROR and PRR dropped to 19.04 (95% CI: 17.02–21.30) and 16.53 (95% CI: 14.78–18.50), respectively. Serious outcomes such as disability (18.7%), life-threatening events (12.9%), and death (7.5%) were also noted. **Conclusions**: The upward trend in suicidality-related safety signals among young male users since 2019, which peaked in 2024, reflects emerging safety concerns among finasteride users, reinforcing the need for pharmacovigilance. Collaborative action among healthcare professionals, regulatory authorities, and pharmaceutical companies, along with clear warnings and mental health assessments before and throughout finasteride therapy, can mitigate potential psychiatric risks and enhance patient safety.

## 1. Introduction

Finasteride, a widely prescribed 5α-reductase inhibitor, is approved for the treatment of benign prostatic hyperplasia (BPH) [1] and androgenetic alopecia (also known as pattern baldness) [2]. In 2021, the global finasteride market was valued at approximately USD 100 million and is projected to grow to around USD 128 million by 2032 [3], primarily driven by its increasing use in treating androgenetic alopecia. This progressive dermatological condition mainly affects men. As a potent inhibitor of the Type 2 5α-reductase isoenzyme, finasteride reduces the levels of dihydrotestosterone (DHT), thereby slowing hair loss and alleviating the symptoms of benign prostatic hyperplasia (BPH) [4]. A study indicates that finasteride reduces scalp DHT levels by approximately 70%, thereby preventing hair loss [5].

Despite its therapeutic benefits, concerns have been raised about its safety profile. Early evidence linked the use of finasteride to sexual dysfunction [6], and more recently, increasing evidence has associated its use with suicidality, depression, and anxiety. Suicidality is a progressive condition which begins with ideation, followed by planning, and can culminate in an attempt, often prompted by psychological distress [7,8].

Growing evidence links finasteride to psychiatric outcomes, particularly in younger people. Nguyen et al. examined VigiBase, the World Health Organization’s global database of individual case safety reports. They found that younger men taking finasteride for androgenetic alopecia had a higher rate of mood-related symptoms like depression and anxiety. The data implies that the psychosocial burden associated with hair loss, combined with the pharmacological effects of finasteride, may contribute to psychological discomfort [9]. A study conducted on a French population revealed that finasteride did not elevate the risk of self-harm when compared to dutasteride, but individuals with a prior history of mood disorders had a higher likelihood of psychiatric complications [10].

However, the issue remains complex as not all studies have found a definitive link between 5α-reductase inhibitors and the risk of suicidality. For instance, a study in 2017 by Welk et al. found no significant increase in suicide among older men taking these medications. The study did observe a higher risk of self-harm and depression, especially during the first 18 months of treatment [11]. This suggests that while the link to suicide might not be straightforward, these medications might still pose mental health risks in specific populations [12].

In response to the mounting and conflicting evidence, regulatory agencies have begun to reassess the safety of finasteride. In 2024, the European Medicines Agency (EMA) launched a safety review of finasteride to better understand the connection between the medication and suicidal thoughts [13]. Similarly, the UK’s Medicines and Healthcare Products Regulatory Agency (MHRA) has recommended revising product labels and including patient safety cards [14].

As evidence suggests links between finasteride and psychiatric adverse effects, a comprehensive understanding of the suicidality risks with its use has become increasingly important. Furthermore, the widespread availability of finasteride via telemedicine and e-commerce platforms has facilitated access, which can lead to unsupervised use, especially among younger individuals who are unaware of psychiatric risks [15]. To address these concerns, real-world evidence and post-marketing safety surveillance are valuable tools for detecting safety signals. The U.S. Food and Drug Administration Adverse Event Reporting System (FAERS) is a comprehensive repository of adverse events reports, which can provide valuable insights into safety concerns. Based on recent reports, we hypothesize that finasteride use could be associated with an increased risk of suicidality-related adverse events, particularly among younger males using it for androgenetic alopecia. Therefore, this study aims to analyze FAERS data to identify the risk of suicidality associated with finasteride, utilizing disproportionality pharmacovigilance analyses, and ultimately contribute to improved patient safety.

## 2. Results

A total of 1566 finasteride-related suicidality adverse event reports (AERs) were identified between 2015 and 2024 (Table 1).

**Figure 1:**
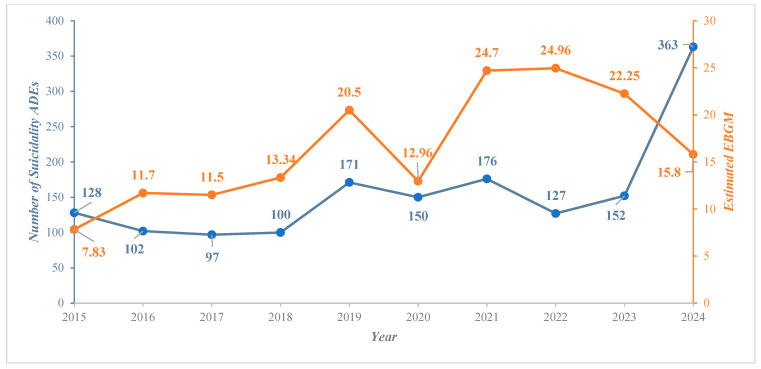
The trend of finasteride-related suicidality adverse event reports with corresponding empirical Bayes geometric mean (total reports/year and EBGM/year).

During the study period, the number of reports remained relatively stable from 2016 to 2018, followed by an increase with some fluctuations in the years 2019 to 2023, and reached its peak in 2024, with a total of 363 reports (Figure 1).

**Figure 2:**
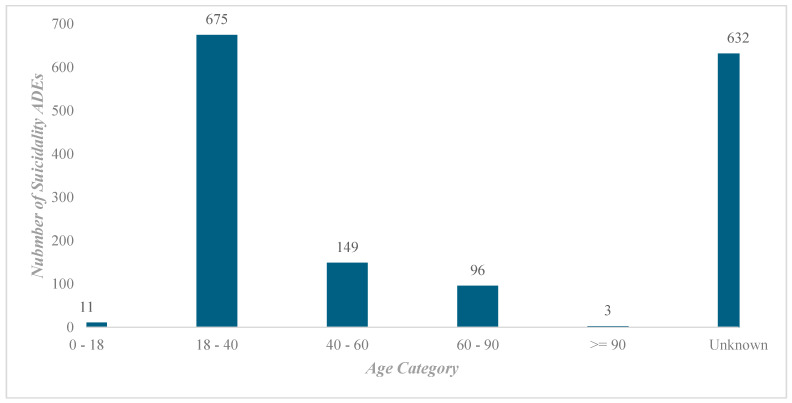
Finasteride-related suicidality adverse event reports stratified by age (years).

The majority (87%) of the patients reported were men, while 1% were women and 12% were of unknown gender. In Figure 2, the age categories of the patients showed an interesting pattern, with 43.1% between the ages of 18–40 years old (*n* = 675), followed by 9.51% between 40 and 60 years old (*n* = 149) and 6.32% over the age of 60 years (*n* = 99).

**Figure 3:**
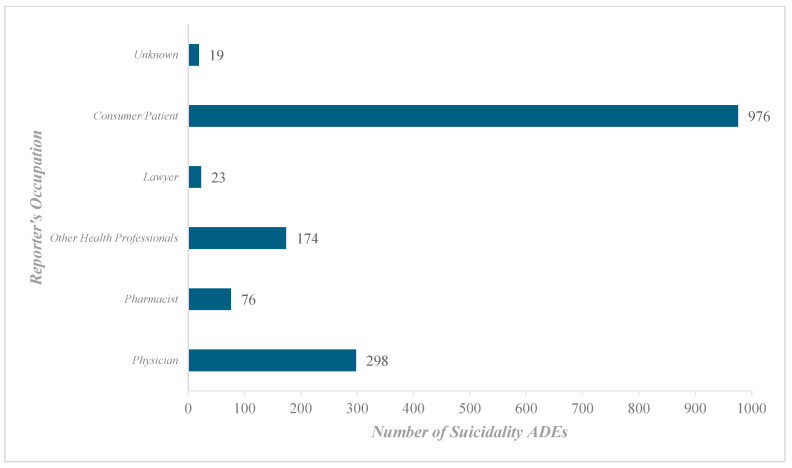
Finasteride-related suicidality adverse event reports stratified by the reporter’s occupation.

In addition, the reporting characteristics of these finasteride-related adverse events of suicidal ideation (AERs) were intriguing. In Figure 3, most reporters were consumers/patients, contributing a staggering 62.32% of the reports (*n* = 976), followed by physicians (*n* = 298, 19.03%), other health professionals (*n* = 174, 11.11%), pharmacists (*n* = 76, 4.85%), and finally lawyers (*n* = 23, 1.47%).

**Figure 4:**
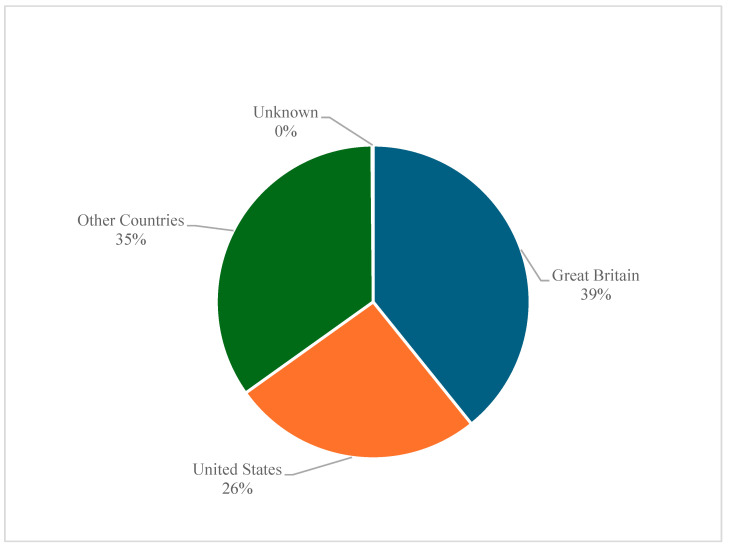
Finasteride-related suicidality adverse event reports stratified by the country of the reporter.

Moreover, the majority (39.21%) of the reports originated from Great Britain, followed by 25.93% from the United States and 34.74% from various other countries (Figure 4).

**Figure 5:**
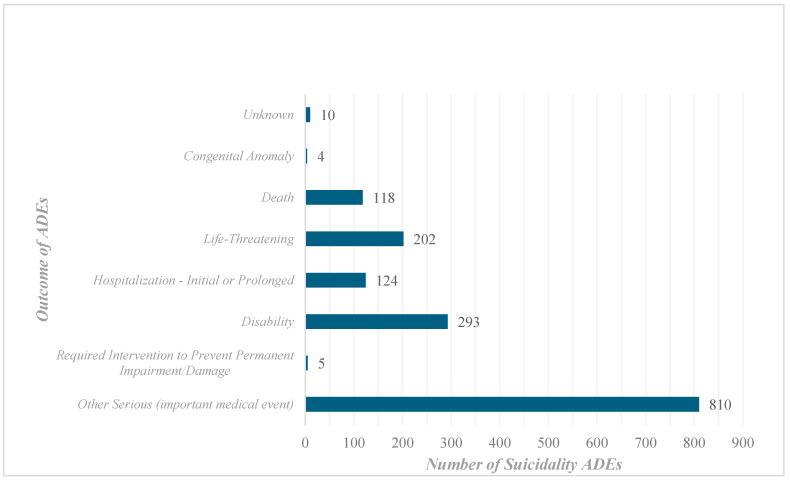
Outcomes of the finasteride-related suicidality adverse event reports (ADEs).

The reported outcomes of these AERs were identified. Over half of the reports (51.72%) had an outcome classified as a serious or important medical event, 18.71% as disability, and 12.9% as life-threatening (Figure 5).

**Figure 6:**
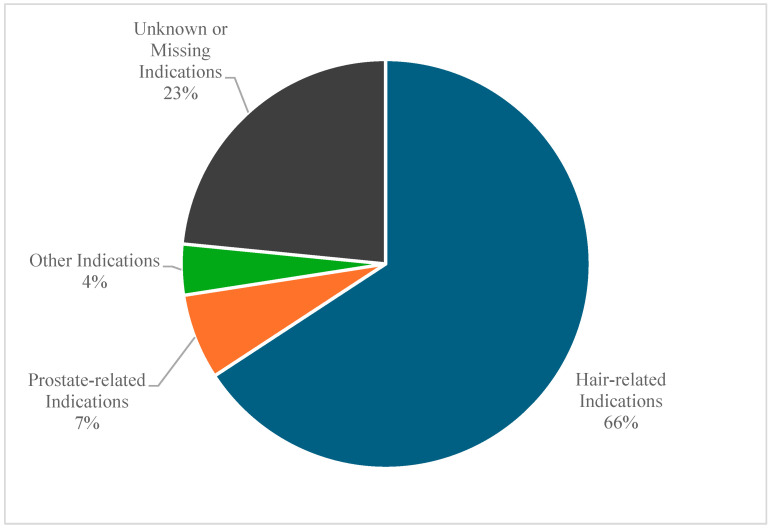
Finasteride-related suicidality adverse event reports stratified by indication.

On the other hand, the reported indications of finasteride in these reports had an interesting pattern. Figure 6 shows that the majority (66%) of the reported indications were hair loss-related, 7% were related to prostate abnormalities, 4% for other indications, and 23% were unknown or missing.

Significant signals of finasteride-related suicidality were observed throughout the study period and across all disproportionality metrics (Table 2).

The highest signal was observed in 2022, with an estimated reported odds ratio (ROR) of 34.64 (28.36–41.88, 95% confidence interval (CI)), followed by a ROR of 33.72 (28.52–39.85, 95%CI) in 2021, and a ROR of 30.53 (25.50–36.56, 95%CI) in 2023. The lowest signal was observed in 2015, with a ROR of 8.76 (7.30–10.50, 95%CI). Furthermore, the risk results obtained using the empirical Bayes geometric mean (EBGM) and the information component (IC) showed a similar pattern and were statistically significant. Once more, the highest risk signal was observed in 2022, with an EBGM of 24.96 (IC = 4.64), followed by an EBGM of 24.70 (IC = 4.62) in 2021, an EBGM of 22.25 (IC = 4.48) in 2023, and an EBGM of 20.50 (IC = 4.36) in 2019. Although the number of reports drastically increased in the year 2024 (*n* = 363), the safety signals were relatively lower compared to those of 2022, 2021, 2023, and 2019, respectively, with a ROR of 19.04 (17.02–21.30, 95%CI), an EBGM of 15.80, and an IC of 3.93.

## 3. Discussion

The study analyzed FAERS case safety reports on suicidality-related signals associated with finasteride, giving additional insights into the potential mental health risks associated with its use amid limited research and rising regulatory concerns.

Our findings indicate that 87.23% of finasteride users are males, and nearly half are between 18 and 40 years old. This gender disparity aligns with the primary indications for finasteride, including androgenetic alopecia and prostate-related conditions, both of which predominantly affect men. The age distribution further suggests that younger individuals were the most frequent users, likely seeking treatment for androgenetic alopecia. In contrast, only 15.83% of users were above 40 years old, indicating limited use among older individuals for BPH. Also, FAERS does not consistently provide dosing details, and as most reports were for hair loss treatment, it could plausibly be the 1 mg/day dose. Thus, the observed psychiatric adverse event patterns are most likely attributable to the lower-dose regimen used for cosmetic purposes rather than the 5 mg/day dose for BPH. These findings are consistent with those of Nguyen et al. (2021), who analyzed Vigibase data and found that 98.9% of finasteride users were male, with 70.9% aged between 18 and 44 years [9]. The similarity between our results and those of Nguyen et al. reinforces the notion that younger men are likely to be finasteride users, mainly for hair loss management.

Psychological distress associated with hair loss may prompt individuals to seek treatment [16,17]. Younger men experiencing hair loss may be particularly concerned about societal expectations and their body image [18], which influence their decision to pursue treatment. Androgenetic alopecia is more prevalent among Caucasian men, affecting approximately 30% by their mid-30s compared to lower rates in the Asian and African populations [19,20], suggesting that white men exhibit higher treatment-seeking behavior due to social and psychological factors.

The geographical distribution of reports shows increased reporting rates from Great Britain (39.21%) and the United States (25.93%), likely due to awareness through advocacy groups, well-developed pharmacovigilance systems, and higher prescription rates. Organizations such as the Post-Finasteride Syndrome Foundation and Citizen Petition groups have raised public and regulatory awareness, persuading the FDA to include warnings in advertisements or restrict the use of finasteride [21,22,23]. Similarly, the October 2024 announcement by the Pharmacovigilance Risk Assessment Committee (PRAC) of the European Medicines Agency to comprehensively review all finasteride-containing products and recommend appropriate regulatory actions [24] underscores the importance of drug safety monitoring in promoting awareness of adverse event reporting. While some countries are at the forefront in reporting safety signals, regional disparities exist, exposing gaps in global pharmacovigilance.

The analysis of finasteride-related suicidality signals in the data extracted from 2015 to 2024 reveals fluctuating trends, with an overall upward trajectory from 2019 onwards and a surge in 2024 (363 reports). Multiple factors may contribute to this increase. As finasteride is available as a prescription-only medication, higher reporting in recent years could be suggestive of increased utilization and evolving prescribing patterns. Awareness driven by advocacy efforts may have stimulated reporting, resulting in a higher number of documented cases. Also, regulatory policy changes or improvements in reporting mechanisms might have led to increased reporting. Furthermore, genetic predisposition among new user populations might also contribute to an increased risk of suicidality [25,26]. Beyond conventional reasons, human behavior is influenced by various contextual factors. For instance, suicidality has risen globally during and after the COVID-19 pandemic [27,28,29], as pandemics can cause psychological distress [30,31]. The upward trend in suicidality-related reports since 2019 suggests that external stressors may contribute to the observed pattern. However, this study does not establish a causal link between COVID-19 and the risk of suicidality associated with finasteride, necessitating further investigation.

Suicidal ideation may arise from mood changes induced by finasteride, with evidence suggesting a possible genetic link between 5α-reductase inhibition and depression [26]. Given that depression can be a trigger for suicide [10], concerns over the psychiatric outcomes of finasteride are growing [32]. The analysis of finasteride-related adverse events shows a concerning pattern, with significant medical events, including fatalities. In one-half of the reports, “other serious medical events” were documented, suggesting a broad range of psychiatric and sexual disturbances. Our findings agree with existing studies acknowledging sexual dysfunction, mood disturbances, and thoughts of self-harm as possible consequences of finasteride use [11,26,33]. Functional impairment and overall quality of life can also be compromised as disability and hospitalization were also reported. Furthermore, severe outcomes, such as life-threatening situations (12.9%) and fatalities (7.54%), challenge the prevailing perception that finasteride is a medication with minimal risk [34].

Recent research suggests that pharmaceuticals which alter steroid hormone pathways, such as finasteride, may significantly impact cerebral chemistry [26]. The neurobiological mechanisms linking finasteride to psychiatric disturbances such as depression, anxiety, and suicidality are multifactorial and increasingly supported by both clinical and preclinical evidence. Central to this association is the inhibition of 5α-reductase, an enzyme essential not only for converting testosterone into DHT but also for producing neurosteroids, such as allopregnanolone [10]. Allopregnanolone is a potent positive allosteric modulator of the GABA_A_ receptors, playing a critical role in inhibiting excitatory neurotransmission in the brain [35,36,37]. Reduced levels of allopregnanolone due to finasteride use have been shown to impair GABAergic signaling, thereby reducing neuronal inhibition and potentially contributing to mood instability and suicidal ideation [10,35,36,37].

Moreover, GABAergic inhibition can lead to glutamatergic overactivation of the NMDA receptors, contributing to excitotoxicity, which is implicated in major depressive disorder and suicidal behavior [9,26,38]. Finasteride-induced suppression of neurosteroid synthesis may also dysregulate serotonergic pathways, impairing both serotonin release and receptor sensitivity, further contributing to mood dysregulation [25].

Additionally, epigenetic changes and androgen receptor upregulation, as observed in preclinical studies, suggest that long-lasting effects on brain function may persist even after discontinuation [26,39]. Individuals with genetic polymorphisms in the SRD5A2 gene, which encodes 5α-reductase, may be particularly susceptible to neurosteroid depletion and its neuropsychiatric sequelae [40].

Emerging evidence also suggests interactions with dopaminergic systems, which play a crucial role in motivation and emotional regulation. Disruption in dopamine signaling due to neurosteroid imbalance could further explain reports of anhedonia, emotional blunting, and suicidal ideation in finasteride users [26,39]. Given the influence of hormonal, neurochemical, and genetic factors, it is evident that the psychiatric effects of finasteride are not merely incidental but likely the result of a complex neuroendocrine disruption. This emphasizes the need for personalized risk assessments and routine mental health monitoring, particularly in younger men using the drug for esthetic reasons. Furthermore, chronic stress associated with the COVID-19 pandemic can suppress neurosteroid biosynthesis [41]. When combined with finasteride-induced neurosteroid depletion, it may cause emotional dysregulation [28]. Disruption in neuroactive steroid pathways might impair neural circuits for emotional processing and impulse control, lowering the threshold for suicidal behavior [10,42].

Our analysis followed the REporting of a Disproportionality Analysis for DrUg Safety Signal Detection Using Individual Case Safety Reports in PharmacoVigilance (READUS-PV) guidelines [43]. Statistical measures, including the ROR, PRR, EBGM, and IC, were used to assess the relationship between finasteride exposure and the risk of suicidality. Our findings indicate a substantial increase in suicidality-related adverse drug events (ADEs) since 2019, a trend that aligns with earlier work [11]. Finasteride-related suicidality signals escalated 2.84-fold between 2015 and 2024, with the number of reported cases nearly doubling from 2019 to 2024 (171 reports to 363 reports). The ROR for suicidality signals in 2021 was 33.72, increasing to 34.64 in 2022 and slightly declining to 30.53 in 2023. However, despite the rise in absolute case numbers, the ROR and PRR values showed a downward trend, decreasing from 27.51 and 27.96 in 2019 to 19.04 and 16.53 in 2024, respectively. The observed decline could be due to an overall increase in the total number of ADEs recorded in the database, which dilutes the risk estimate. Based on the EBGM estimate, our study findings indicate that reports of suicidality associated with finasteride doubled between 2015 and 2024 (7.83 vs. 15.8). Further, positive IC value for finasteride suggests that the frequency of drug–ADR pair reporting exceeds the expected rate, considering all reports in the database. Nearly two-thirds of the reports came from consumers, highlighting the importance of patient awareness in recognizing and reporting side effects. For example, in the UK, the Medicines and Healthcare Products Regulatory Agency (MHRA) has introduced a patient alert card within the packaging to inform users about potential risks [44]. An earlier study also reported higher reporting rates from finasteride consumers [33]. Although self-reporting serves as a valuable tool for documenting genuine experiences, dissemination of information can also trigger a nocebo effect [45,46], as evidenced by the overreporting of sexual dysfunction with the use of finasteride. On the contrary, our findings reveal minimal reporting by physicians and pharmacists. This inconsistency calls for initiatives to improve awareness among healthcare professionals, as they could play a pivotal role in identifying and documenting psychiatric adverse events.

The implications of our study’s findings are significant for both clinical practice and public health policy. Healthcare providers should implement a proactive strategy in monitoring patients prescribed finasteride, especially younger males using the medication for esthetic reasons. Routine mental health assessments should be established as a standard procedure, accompanied by a discussion regarding potential risks before the commencement of treatment [14]. Regulatory agencies must strengthen pharmacovigilance frameworks, update labeling with explicit warnings about suicidality risk and support independent research to establish causal relationships. Also, public education initiatives should raise awareness of possible psychiatric side effects, encourage early recognition, pursue timely assistance, and reduce the stigma surrounding mental health [14].

**Strengths and limitations**

One of the notable strengths of this study is the use of the FAERS dataset, which spans nearly a decade (2015–2024), allowing for a longitudinal analysis of suicidality-related adverse event reports associated with the use of finasteride. The dataset showed a marked increase in suicidality-related reports in 2019, with a peak in 2024 (363 reported cases). This trend coincides with the rise in global mental health challenges during and following the COVID-19 pandemic [46,47,48], thus providing a clue that external stressors may amplify the psychiatric effects of finasteride.

The study used disproportionality analysis methods, including ROR, PRR, EBGM, and IC, to enhance the robustness and validity of the findings. FAERS is a U.S.-based system that receives adverse event reports from around the world, providing a global perspective on the safety profile of finasteride.

An additional key finding of the study was the identification of younger users (ages 18–40) as the most affected group, likely due to cosmetic purposes, such as hair loss treatment. When the findings were stratified based on age and indication, it was evident that young users had an increased risk for neuropsychiatric side effects.

While the analysis offers significant insights, it is essential to acknowledge the study’s limitations. The spontaneous reporting systems highlight intrinsic biases, including underreporting, incomplete data, and the lack of denominator information.

This study found that most reports (62.3%) came from consumers, while only 19.03% were reported by physicians and 4.85% by pharmacists. Self-reports are valuable for capturing stigmatized symptoms like mood disturbances, but they lack clinical validation [44,49]. Furthermore, consumer reports may be emotionally driven, mainly when influenced by media attention or litigation [21,26]. The rise in suicidality-related reports may, in part, be attributed to notoriety bias, where specific adverse events may be disproportionately reported due to regulatory action. In the case of finasteride, the activities of the Post-Finasteride Syndrome Foundation and its lawsuit with the FDA [49,50], as well as widespread public discourse on social media, may have contributed to stimulated reporting. When external narratives prompt individuals, they tend to retrospectively associate their symptoms with the drug and report them [43,51], inflating signal strength without accurately reflecting causality [2,22,32,49,52]. This pattern is consistent with the sharp increase in suicidality-related reports observed in our dataset beginning in 2021 and peaking in 2024.

Underreporting is a limitation of the spontaneous reporting systems, with studies estimating that as few as 1–10% of actual adverse drug events are reported [43]. This implies that the true incidence of finasteride-related suicidality could be higher than documented. The relatively low reporting rate by healthcare professionals, despite their role in diagnosis and pharmacotherapy, might arise from a lack of awareness, time constraints, or the assumption that adverse events are already known. They may also be discouraged from reporting due to lawsuits and advocacy campaigns [50,53].

Missing data, such as that regarding age (40.36%), gender (12.2%), and indication (23%) further weakens the robustness of subgroup analyses. Although 43.1% of reports were from individuals aged 18–40, most likely to have used finasteride for androgenetic alopecia, missing age data limited our ability to stratify psychiatric risk across life stages and reduced the generalizability of this finding [49]. Similarly, missing gender data constrained the ability to assess sex-based differences in neuropsychiatric susceptibility despite the male predominance linked to the use of finasteride [54]. Missing indication data hindered the assessment of dose-related risks, as 1 mg/day is used for AGA and 5 mg/day for BPH, limiting our ability to distinguish between esthetic and therapeutic contexts [55]. Such data gaps are inherent to spontaneous reporting systems, such as FAERS, which rely on voluntary reporting [43]. To avoid bias, we refrained from overinterpretation and disclosed missing data. Future research utilizing structured databases, such as electronic health records (EHRs) or claims data, could provide a more robust and reliable analysis.

Another limitation is the inability to adjust for potential confounders, such as existing comorbidities or concurrent medications, which may influence the outcomes. The reported data lacked details on psychiatric illness history, concomitant use of psychotropic medications, and comorbidities. The lack of this information undermines our ability to determine if suicidality signals are directly attributable to finasteride use or influenced by these variables.

Although the ROR and PRR values for 2024 are lower compared to previous years, the absolute number of reports (363) is substantial, reflecting the shortcomings of conventional disproportionality metrics in accurately reflecting absolute risk levels, particularly in rapidly escalating case numbers, a pattern referred to as “dilution effect”. As the total volume of reports in the FAERS increases, the background frequency of all adverse events increases, potentially reducing the relative disproportionality for any specific drug–event pair, even when absolute numbers rise. Therefore, integrating disproportionality metrics with other epidemiological approaches or machine learning models can better predict and identify at-risk subpopulations [56,57], providing a more accurate estimate of drug safety.

To address these limitations and enhance causal inference, we recommend future studies which use cohort or case–control designs to compare exposed and unexposed groups [10,11], and the utilization of electronic health records, which can provide clinically validated data and integrate genomic data (SRD5A2 variants) to assess individual susceptibility [40,41].

To increase healthcare professional participation and improve data quality, we propose launching educational campaigns, pharmacovigilance-based continuing education programs [9], and embedding ADR reporting tools within electronic medical records to facilitate clinical documentation [58]. The use of standardized discharge forms, which include queries on ADR, could be beneficial in increasing the number of ADR reports [59]. Furthermore, incentivization could encourage routine reporting of psychiatric ADRs.

## 4. Materials and Methods

This study utilized data from the FAERS, reported from 2015 to 2024. The FAERS collects reports of adverse events and medication errors related to both regular medicinal products and biologics from various sources, including healthcare professionals, pharmaceutical manufacturers, lawyers, and patients. The database is freely accessible on the FDA website at https://open.fda.gov/data/faers/ (5 March 2025) and is updated quarterly. Although FAERS is a U.S.-based database, it receives adverse event reports from around the world. Its extensive size and global scope make it well-suited for analyzing spontaneous reporting data. Notably, the database contains anonymized information, ensuring the privacy of individuals.

The FAERS database comprises seven file types: Demographic and Administrative Information (DEMO), Drug Information (DRUG), Adverse Events (REAC), Patient Outcomes (OUTC), Report Sources (RPSR), Start and End Dates for Reported Drugs (THER), and Indications for Use (INDI). Each type of database is categorized based on the reporters, such as physicians, pharmacists, other healthcare professionals, consumers, or lawyers. Outcomes are classified as death, life-threatening, hospitalization, disability, congenital anomaly, or requiring intervention to prevent permanent damage. The reporter’s country reflects the origin of the latest report version, which may be within or outside the United States, encompassing any country that submits adverse drug reactions to FAERS. Patient age is recorded as a numeric value at the time of the event [60,61].

A unique identifier, “primaryid”, was used to link all datasets for this study. The U.S. Food and Drug Administration (FDA) requires pharmaceutical manufacturers to report adverse drug reactions (ADRs) associated with their products. Healthcare professionals and consumers/patients are also encouraged to submit ADR reports, regardless of their location [62,63,64].

Reported data were retrieved from the FAERS database between 2015 and 2024 to investigate suicidality concerns associated with the use of finasteride. The FAERS database employs the Medical Dictionary for Regulatory Activities (MedDRA) coding system, which uses preferred terms (PT) to classify adverse events (AEs). Using PT, multiple search terms, including “suicidality,” “suicide,” and “suici,” were employed to capture all suicidality-related reports. The research team further verified the outputs to ensure that only terms related to suicidality were included.

The study focused on reports involving the main concerns that have been raised regarding finasteride’s potential psychiatric side effects, such as depression and suicidality, which manifest as suicidal ideation, attempts, or complete suicide. Reports were retrieved using the “drugname” and “prod_ai” variables in the database. Additionally, only reports where the medication was listed as the primary suspected (PS) cause of the adverse event were included, minimizing bias from secondary suspected (SS) or concomitant medications. Duplicate reports were identified and removed using the “primaryid,” event date (event_dt) and “pt” variables.

**Statistical analysis**

A pharmacovigilance case/non-case disproportionate analysis was conducted to evaluate the risk of potential psychiatric side effects such as depression and suicidality associated with finasteride. Cases were defined as potential psychiatric event reports linked to finasteride, while non-cases included all other adverse event reports for the exposure of interest (i.e., finasteride).

Descriptive statistics were calculated for all items recorded within the studied population. Furthermore, both Bayesian and traditional statistical methods were employed to assess potential safety concerns. A 2 × 2 contingency table was used to analyze four key metrics. The table included four components: “a” (cases for the studied medications), “b” (non-cases for the studied medications), “c” (cases for other medications), and “d” (non-cases for other medications) (Table 3) [61].

A dual-method approach was adopted to enhance the robustness of the analysis and reduce the risk of erroneous findings. The disproportionality metrics computed included the reporting odds ratio (ROR), proportional reporting ratio (PRR), empirical Bayes geometric mean (EBGM), and information component (IC). A safety signal was indicated by an ROR above one, a PRR of two or higher, an EBGM exceeding two, or an IC value greater than zero [64,65].

To ensure reliability, significant signals had to meet the criteria across all four metrics. This approach minimized the likelihood of false associations. All statistical analyses were performed using R software (version 4.2.2) and R Studio (Version 2024.04.2+764).

## 5. Conclusions

This study has identified growing safety signals related to suicidality associated with finasteride, which intensified in 2019 and peaked in 2024, particularly among young men. The increasing usage also highlights the need for collaborative efforts among stakeholders to mitigate risks and ensure patient well-being. Additionally, reporting from healthcare professionals, which is currently found to be low, is crucial for accurate safety assessments. As the safety profile of finasteride continues to evolve, sustained surveillance and scrutiny will be essential to ensure its safe use.

## Figures and Tables

**Table 1:** Patients’ characteristics from the finasteride-related suicidality adverse event reports.

Characteristics	Frequency	Percentage
**Patient’s Gender**	***n***	**%**
Male	1366	87.23
Female	9	0.57
Unknown	191	12.2
**Patient’s Age (years)**		
0–17	11	0.7
18–39	675	43.1
40–59	149	9.51
60–89	96	6.13
≥90	3	0.19
Unknown	632	40.36
**Reporter’s Occupation**		
Physician	298	19.03
Pharmacist	76	4.85
Other Health Professionals	174	11.11
Lawyer	23	1.47
Consumer/Patient	976	62.32
Unknown	19	1.21
**Reporter’s Country**		
Great Britain	614	39.21
United States	406	25.93
Other Countries	544	34.74
Unknown	2	0.13
**Outcome**		
Other Serious (important medical event)	810	51.72
Required Intervention to Prevent Permanent Impairment/Damage	5	0.32
Disability	293	18.71
Hospitalization—Initial or Prolonged	124	7.92
Life-Threatening	202	12.9
Death	118	7.54
Congenital Anomaly	4	0.26
Unknown	10	0.64
**Total**	**1566**	**100**

Outcome: the reported consequence of the finasteride-related suicidality event, which should be interpreted carefully as it does not necessarily entail causality. Unknown: missing data.

**Table 2:** Risk of suicidality * and the use of finasteride during 2015 to 2024.

Year	Number of Suicidality ADEs	Reported ADEs with the Drug of Interest	ROR (95% CI)	PRR (95% CI)	EBGM	IC
**2015**	128	1549	8.76 (7.30–10.50)	8.11 (6.77–9.73)	7.83	2.97
**2016**	102	982	13.77 (11.21–16.92)	12.45 (10.13–15.30)	11.7	3.55
**2017**	97	612	14.33 (11.53–17.82)	12.22 (9.83–15.20)	11.5	3.52
**2018**	100	740	16.38 (13.26–20.24)	14.3 (11.58–17.67)	13.34	3.74
**2019**	171	817	27.51 (23.22–32.58)	21.96 (18.54–26.01)	20.5	4.36
**2020**	150	1034	15.74 (13.23–18.72)	13.61 (11.43–16.18)	12.96	3.7
**2021**	176	831	33.72 (28.52–39.85)	26.79 (22.66–31.66)	24.7	4.62
**2022**	127	640	34.64 (28.36–41.88)	27.82 (22.90–33.81)	24.96	4.64
**2023**	152	704	30.53 (25.50–36.56)	24.15 (20.17–28.92)	22.25	4.48
**2024**	363	2614	19.04 (17.02–21.30)	16.53 (14.78–18.50)	15.8	3.98

* Suicidality includes suicidal thoughts, suicidal ideation, suicide attempts, depression, suicidal mood, and complete suicide. ROR—reporting odds ratio; PRR—proportional reporting ratio; EBGM—empirical Bayes geometric mean; IC—information component.

**Table 3:** Contingency table for disproportionality analyses.

	* Adverse Event Reports of Interest *	* Other Adverse Event Reports *
* Drug of interest *	a	b
* All the other drugs in the database *	c	d

## Data Availability

The FDA Adverse Event Reporting System (FAERS) quarterly data extract files are publicly available and can be obtained from: https://fis.fda.gov/extensions/FPD-QDE-FAERS/FPD-QDE-FAERS.html (accessed on 4 October 2024).

## Abbreviations

The following abbreviations are used in this manuscript:

EBGM	Empirical Bayesian Geometric Mean
IC	Information Component
ROR	Reporting Odds Ratio
PRR	Proportional Reporting Ratio
BPH	Benign Prostatic Hyperplasia
DHT	Dihydrotestosterone
FAERS	Food and Drug Administration Adverse Event Reporting System
AERs	Adverse Event Reports
